# Impact of lived experiences of people with disabilities in the built environment in South Africa

**DOI:** 10.4102/ajod.v9i0.518

**Published:** 2020-08-06

**Authors:** Victor McKinney, Seyi L. Amosun

**Affiliations:** 1 Department of Health and Rehabilitation Sciences, University of Cape Town, Cape Town, South Africa

**Keywords:** people with disabilities, lived experiences, built environment, challenge, South Africa

## Abstract

**Background:**

In spite of legislations and policies to ensure an inclusive society in South Africa for the accommodation of people with disabilities, there are reports that they still struggle to move freely within society.

**Objectives:**

As part of a larger qualitative exploratory study on the preparation of undergraduate civil engineering students in a local university to contribute to the development of an inclusive society, this article seeks to understand the impact of the lived experiences of people with disabilities in their interaction with the built environment.

**Method:**

Four persons with disabilities, considered to be knowledgeable about South African legislations relating to disability, were purposely selected to each share one specific experience whilst interacting with the built environment. The transcribed texts of the interviews were analysed by using the phenomenological–hermeneutic method.

**Results:**

The participants exhibited strong desires to participate in society. However, the sense of loss of control and independence as they encountered challenges in the built environment changed the euphoria to disempowerment, rejection, anger and despondency. In spite of their experiences, participants expressed a commitment towards overcoming the challenges encountered in the broader interest of people with disabilities.

**Conclusion:**

A deeper understanding of the impact of the experiences of people with disabilities when they participate within the built environment in South Africa revealed a broad spectrum of negative emotions, which may impact the quality of life and well-being of the participants.

## Introduction

In an attempt to understand the contextual parameters that would impact disability inclusion in South Africa, this article explores the lived experiences of some persons with disabilities with regard to inclusion in a built environment that is assumed, based on available government’s documentations, to align with international and national disability-related policies and legislations (Department of the Presidency [DOP] 2013; Department of Women, Children and People with Disabilities [DWCPD] 2013). These include the United Nations Convention on the Rights of Persons with Disabilities (UNCRPD) (United Nations [UN] 2006), and at national level, the Integrated National Disability Strategy (INDS) (Office of the Deputy President [OSDP] 1997), the National Development Plan 2030 (NDP) (DOP 2013) and the White Paper on the Rights of Persons with Disabilities (WPRPD) and its accompanying implementation matrix (Department of Social Development [DSD] 2016). The research presented in this article was part of a larger study, which explored the readiness of undergraduate civil engineering students at a local university in South Africa to contribute to the development of an inclusive society that accommodates people with disabilities (McKinney 2016).

For the purpose of this article, the definition of the built environment consists of ‘all buildings, spaces, and products that are created or modified by people’ (Smit et al. 2016:197). Globally, the estimated number of people living with some form of disability is one billion, with approximately 190 million living with severe disabilities (World Health Organization [WHO] 2011). In South Africa, approximately 10% of the population live with a disability (Statistics South Africa 2011), although there have been fluctuations in the recorded prevalence (Maart et al. 2007; Schneider 2009; Sing 2012). Persons with disabilities represent a fair portion of the world population, and they form ‘a diverse group who share the experience of living with significant limitations in functioning and, as a result, often experience exclusion from full participation in their communities’ (Krahn, Walker & Correa-De-Araujo 2015:198). This social exclusion creates painful experiences that have short- and long-term detrimental effects on the well-being of affected individuals (Delbosc & Currie 2011; Klompas & Ross 2004; Krahn et al. 2015; Tobias & Mukhopadhyay 2017). It is therefore helpful to understand the experiences of persons with disabilities in their attempt for inclusion in all spheres of the societies they live in (Hammel et al. 2015). This will align with the World Bank’s goal of building partnerships with the world’s leading disability groups to advance social and economic inclusion (World Bank 2016).

Although there is much discourse on what constitutes an inclusive society, there are few definitions. The World Summit for Social Development in Copenhagen in 1995 defined an inclusive society as a ‘society for all in which every individual, each with rights and responsibilities, has an active role to play’ (UN 1995). The UN (2008) later emphasised that such an inclusive society must be:

[*B*]ased on respect for all human rights and fundamental freedoms, cultural and religious diversity, social justice and the special needs of vulnerable and disadvantaged groups, democratic participation and the rule of law. (p. 8)

Based on these underlying principles of an inclusive society, the Centers for Disease Control and Prevention envisage that disability inclusion entails including people with disabilities in everyday activities and encouraging them to have roles similar to their peers who do not have a disability. This involves more than simply encouraging people; it requires making sure that adequate policies and practices are in effect within a community or organisation.

‘Inclusion should lead to increased participation in socially expected life roles and activities’ (Centers for Disease Control and Prevention 2016:para. 1).

In summary, disability inclusion is conceptualised as a process of identifying, understanding and breaking down the barriers to participation and belonging rather than a fixed state (Armstrong, Armstrong & Spandagou 2011; Mattevi et al. 2012). Internationally, a wide range of research has covered the experiences of people with disabilities interacting with the built environment. Much has focussed on individual impairments such as those with visual, hearing or mobility impairments and their experiences including navigating buildings (Legge et al. 2013), travel activities (Poria, Reichel & Brandt 2011), higher education and employment (Byrne 2014; Kramer 2008) and transport systems (Pyer & Tucker 2017). More comprehensive studies investigated how people with disabilities perceived the role of the built environment in their participation in various aspects of daily life (Hammel et al. 2015). Literature categorises the barriers to disability inclusion as attitudinal, environmental and institutional (Harpur 2012). For an inclusive society to develop, it is imperative that people with disabilities have access to their environment (UN 2006) as this allows for a platform where disability may be celebrated in its diversity, as opposed to being excluded as something different, which is currently a common experience of people with disabilities (Clarke et al. 2011; WHO 2011).

With South Africa’s transition to democracy in 1994, the constitution held up the promise of a better life for all South Africans, raising the aspiration for an inclusive society that accommodates persons with disabilities. The conceptualisation of disability evolved for the better in the country, with the adoption of the social model of disability presented in the UNCRPD (Harpur 2012). Article 9 of the UNCRPD specifically calls for the development of an accessible built environment to accommodate people with disabilities (UN 2006). However, unfortunately, it seems that not much change is happening on the ground to enhance the inclusion of persons with disabilities (Amosun & Taukobong 2010; Amosun, Volmink & Rosin 2005; Lucas 2012; Mayat & Amosun 2011). South Africa was the second country to ratify the UNCRPD and incorporated its comprehensive framework (Borg, Larsson & Östergren 2011; Mannan et al. 2012). The UNCRPD comprises of 50 articles that outline the protection of rights and dignity of people with disabilities, addressing all aspects of quality of life and their full participation in society. The WPRPD that was launched by the President of South Africa in 2016 described disability inclusion as follows (DSD 2016):

Inclusion is regarded as a universal human right and aims at embracing the diversity of all people irrespective of race, gender, disability or any other differences. It is about equal access and opportunities and eliminating discrimination and intolerance for all. It is about a sense of belonging: feeling respected, valued for who you are; feeling a level of supportive energy and commitment from others so that you can best fully participate in society with no restrictions or limitations. Inclusion implies a change from an ‘individual change model’ to a ‘system change model’ that emphasises that society has to change to accommodate diversity, i.e. to accommodate all people. This involves a paradigm shift away from the ‘specialness’ of people to the nature of society and its ability to respond to a wide range of individual differences and needs. Inclusion is the ultimate objective of mainstreaming. (p. 8)

Although some countries have enacted and enforced specific antidiscrimination legislations as a step towards ensuring social inclusion (Vanhala 2006), South Africa does not yet have specific legislation pertaining to the rights of people with disabilities. However, one may find protection for persons with disabilities in core legislative acts such as *The Constitution* (1996) and *the Promotion of Equality and Prevention of Unfair Discrimination Act* (PEPUDA) (2000), as well as the commitment of government to the inclusion of people with disabilities as expressed in the NDP (DOP 2013). In addition, there are generic policies that are applicable to specific areas of life advocating for the accommodation of people with disabilities (Combrinck & Van Reenen 2011; Dube 2005), such as:

- The *Code of Good Practice on the Employment of People with Disabilities* (2002) – which is essentially an implementation guide for employers to facilitate the employment of people with disabilities.
- *SANS 10400: The application of the National Building Regulations, Part S: Facilities for persons with disabilities* (1987, revised 2011), which is deemed to be compliant with the requirements of the *National Building – Regulations and Building Standards Act, 1977* (Act No. 103 of 1977) (Watermeyer 2014). This policy strives towards the free movement of people with disabilities within the South African built environment.

A couple of studies have explored the impact of the built environment on people with disabilities in South Africa (Adewumi & Allopi 2014; Maart et al. 2007; Napier, Coulson & Matsebe 2006). In addition, previous research has explored the experiences of people with disabilities within the South African context including community stakeholders’ perspectives on the role of occupational therapy (Naidoo, Van Wyk & Joubert 2017), students with disabilities in higher education (Chiwandire & Vincent 2017; Lourens & Swartz 2016), economic vulnerability (Hanass-Hancock et al. 2017) and rehabilitation experiences in rural South Africa (Visagie & Swartz 2016).

However, in spite of the assurances in national policies and legislations, there remains a shortage of information on understanding the impact of the lived experiences of persons with disabilities in accessing the built environment. This article sought to document and gain deeper insight into the lived experiences of persons with disabilities in their desire to access and participate in the built environment.

## Methodology

The exploratory nature of the larger study required a qualitative research approach. Purposive sampling was utilised in selecting four persons with a disability who were considered to be knowledgeable (Merriam 1989) about South African legislations relating to disability. The lead author of this manuscript (V.M.) is a person with disability and is aware that all the participants had been involved in the South African disability sector, personally and professionally, over a number of decades. They had worked across the public and civil sector towards increasing awareness, education and training on disability issues. They had also worked extensively with, and sometimes for, the government and other stakeholders in improving domestic disability policy.

The four participants manifest three of the four types of disability identified by the WHO (2008), namely motor or physical disability, visual disability and hearing impairment (Table 1).

**TABLE 1:** Profile of study participants (*n* = 4).

Type	Disability	Age	Gender	Level of education	Occupation
A	Quadriplegic paralysed from shoulders down	42	Male	Tertiary, PhD	Academic researcher and artist
B	Visually impaired and legally blind	50	Male	University degree	Disability consultant
C	Hard of hearing	38	Female	Tertiary, PhD	Academic researcher and teacher
D	Paraplegic	47	Male	Tertiary, MPhil	Disability consultant

### Data collection

In-depth, semi-structured interviews were conducted separately with each participant, except one. The interview schedule with the person with a disability for the larger study that explored the readiness of undergraduate civil engineering students to contribute to the development of an inclusive society that accommodates people with disability is provided in Appendix 1. Responses to question 10 of the interview schedule provided the data for this article. The in-depth format enabled the researcher ‘to explore fully all the factors that underpin participants answers: reasons, feelings, opinions and beliefs’ (Legard, Keegan & Ward 2003:141). The semi-structured format allowed for an interactive interview where the researcher could probe to gain deeper insight and exploration of the participants’ experiences (Legard et al. 2003).

After some initial questions relating to assessing knowledge about South African policy on disability and accessibility, each participant was asked the following question: ‘Could you please describe an experience that you have had within the South African built environment?’ The question was intentionally broad to avoid any possible bias. This question was followed up with prompts such as: ‘Could you please describe in greater detail….?’ and ‘Could you please explain a bit more about…?’ to get a clear picture and avoid any misunderstanding of the events and the participant’s experience. For the one participant who was not interviewed, he gave permission that related information about his experience could be taken from a newspaper article that was uploaded on his personal blog. Follow-up e-mail correspondence between the participant and one of the authors (V.M.) took place whenever clarity was sought, or for further exploration. The open-ended questions posed to each of the participants offered opportunity to also capture a wide range of emotions in their responses.

### Data analysis

The audio-recorded interviews were transcribed and analysed, by using the phenomenological–hermeneutic method (Davidsen 2013; Lindseth & Norberg 2004). The method has been widely used to interpret the meanings of lived experiences of individuals in different contexts (Angel & Buus 2011; Cassidy et al. 2011; Karlsson, Bergbom & Forsberg 2012). The method involves three key steps. The first step involves a *naive* reading, which provides an initial understanding of the data. The text of the interview is read many times ‘in order to allow the text to speak to us … we become touched and moved by it’ (Lindseth & Norberg 2004:149). The second step involves the *structural analyses* where meaning units are sought within the text.

These meaning units are then condensed (Table 2) and abstracted into themes and subthemes (Tables 3 and 4). The themes were reflected upon by the researchers to ascertain whether they ‘validate or invalidate the naive understanding’ (Lindseth & Norberg 2004:150). The third step is referred to as *comprehensive understanding*, and it entails a summarisation and reflection of all the themes in relation to the context of the study and research question.

**TABLE 2:** Meaning units and condensation example (from the experiences of Participant D, male, 47 years old).

Meaning units	Condensation
So, we get taken to the train, and the feeling sank a bit further when the doorway into the train was a typical narrow door. ‘Can you walk a few steps?’ Hmmm … sink a bit more… ‘No, I am sorry, I cannot walk or even stand at all’ ‘oh…’. Sinking fast now…	Coming to narrow doorway and being asked to walk, realising inaccessibility
The staff look on hopeless…. The manager is getting increasingly irate calls asking why the train is delayed…. They all look suitably embarrassed.	Staff are helpless and embarrassed
My wife wants to get off with me, through her tears. I convince her to stay on, and to enjoy the weekend with our friends.	Convincing upset wife to stay
They have made that mistake – the buttons are not in relief, they are engraved, the tactile must be in relief not engraved. So I argued with them, I got the lift company growling at me, saying ‘no, no, no we are following the strict rules’. They do not listen, sadly. Even when you do tell them chapter and verse, and you can stuff section S (of building regulations) down there.	Stakeholders make mistakes, I argued but they don’t listen and ignore section S
Whether they are aware or not, the message is loud and clear, if you are disabled you don’t belong – that is the message they are sending. And they should be aware, you know. It should not still be this way.	Inaccessibility sends a message that disabled don’t belong, awareness is poor

**TABLE 3:** Sub-themes, themes and main theme from the first structural analysis.

Subthemes	Themes	Main theme
Getting a sinking feeling from staff attitudes towards disability Realising cellblock is inaccessible Being frustrated with other people’s assumptions about disability Having difficulty using inadequate facilities Having disability rights violated	Becoming discouraged from not being able to participate	Feeling rejected and disconnected from society
Losing independence when using inaccessible elevator Getting headache and not being able to communicate Losing the ability to lip-read	Losing autonomy in the built environment	-
Becoming distressed at being excluded Trying not to make everyone feel bad Being embarrassed at losing dignity Having to endure unwanted attention Feeling ashamed for upsetting friends and wife Becoming enraged from being put in unbearable situation Feeling angry at lack of accountability of train company	Being overcome with anger and humiliation	-
Being unable to travel on public transport Having no choice but to leave the inaccessible situation Feeling like giving up Reflecting on the lack of accountability of the construction companies Being disappointed in attitudes of society	Feeling despondent and defeated	-

**TABLE 4:** Sub-themes, themes and main theme from the second structural analysis.

Subthemes	Themes	Main theme
Using elevator successfully with the right device Enjoying the tour Using public transport system Relishing spending time with friends Checking out the environment Trying to adapt to situation Putting health at risk Expecting delivery of human rights	Having the desire to participate in society	Being able to play an active role and contribute to society
Wanting to be independent Having to rely on instinct Appreciating good signage Being frustrated at being dependent	Striving for independence in the built environment	-
Talking to lift company to get accommodations right Working with government to improve accommodation for people with visual impairments Making companies aware of policy Engaging others with challenges of accessibility	Collaborating to improve accessibility	-

The theoretical framework behind the data analysis was predominantly informed by international and domestic policy on disability, specifically Article 9 of the UNCRPD (UN 2006), and Strategic Pillar 1: Removing Barriers to Access and Participation of the WPRPD (DSD 2016), respectively. In other words, the theoretical lens investigates the ability of the participants to fully participate in the South African built environment and play an active role in society, the impact on their quality of life, dignity, health and well-being and how their lived experiences related to current policy on disability.

### Ethical consideration

Permission to carry out the study was granted by the Human Research Ethics Committee (HREC) at the University of Cape Town (ethics approval reference number HREC REF:165/2011). Furthermore, all participants signed a consent form in which the purpose of the study and the rights of the participants was outlined. Permission was also sought and granted from the participants to record the interviews. All data collected were kept in a secure place to which only the researcher had access. There was no link between the interview data (tapes and transcripts) and any identifying data about the research participants.

### Rigour

To ensure trustworthiness, four components, credibility, transferability, dependability and confirmability, were undertaken (Guba, Lincoln, Polit, & Hungler in Graneheim & Lundman 2004). The credibility of the study aimed at avoiding misrepresentation or distortion of the data and was enhanced by prolonged engagement in the field (Bitsch 2005) as well as the process of member checking to verify the responses of participants (Guba & Lincoln 1982). Transferability was addressed through use of purposive sampling and thick description that allows for replication by future researchers conducting similar studies (Shenton 2004). To uphold dependability, all the research processes were documented in detail and kept as an audit trail (Li 2004), which also promotes confirmability (Guba & Lincoln 1982). Confirmability was further enhanced through reflexivity, where the researchers continuously questioned their own predisposition and how this may influence and inform the research (Shenton 2004).

## Findings

A summary of the experiences of each of the four participants is first presented. Participant A is a male quadriplegic paralysed from the shoulders down. He went on a tour to Robben Island in Cape Town, South Africa, where Nelson Mandela spent 18 years of his 27-year prison sentence. He was joined by his wife and her family, who were visiting from the UK, as well as his care assistant. Most of the tour route was accessible, but he encountered a challenge when trying to get into the cellblocks that housed Nelson Mandela’s cell. There were stairs in front of the block, and there was no accessible ramp for wheelchair users.

Consequently, he was stuck outside whilst his wife and her family went into the cellblocks. However, Participant A persisted, and with the aid of some of the tour group members, he descended to a lower level using a makeshift ramp made from two metal beams found nearby. Once descended, he found that he could access all the cellblocks.

Participant B is a man with a visual impairment who described the challenges he encountered when using a hotel elevator that used touch-sensitive buttons, as opposed to the more conventional slightly raised, numbered buttons. He got lost using the elevator after inadvertently activating many touch-sensitive buttons at once, thus triggering a host of independent events. As the lift had no audio, he soon had no idea at which levels the elevator was stopping.

Participant C is a woman, who is hard of hearing, used an express train system in Johannesburg, South Africa to get from the city centre to the airport to catch a flight back home to Cape Town. At one of the stations she needed to change trains and could not find the correct platform because of inadequate signage. She was in danger of running late and missing her flight because she struggled to communicate with the security guards as she tried to lip read them but could not because of the bad lighting at the station. In addition, the loud background noise and poor acoustics on the station made it difficult for her to concentrate.

Participant D is a paraplegic who got the chance to take a weekend trip with his wife and friends on a renowned luxury train in South Africa. Having been assured that the train was accessible, he bought tickets, which although at a reduced price were still very expensive at approximately ZAR 10,000 (US$650 or €590). After enjoying a five-star treatment with champagne with his friends before embarking, it became apparent that the train was in fact not accessible for independent wheelchair users like himself. He tried in vain to use the train’s wheelchair (after getting out of his own custom-built one) and manoeuvre around the trains ‘accessible’ cabin set aside for guests who use wheelchairs. The challenges he encountered included being asked by the train staff to walk a few steps to board the train, use the train’s old inadequate wheelchair, stay in an inaccessible cabin and use the train’s butler every time he wanted to get in and out of bed or use the bathroom. Eventually, he had to return home alone, saying goodbye to his tearful wife and upset friends whom he persuaded to carry on with the journey so as not to miss the once-in-a-lifetime opportunity.

An understanding of the impact of the narrated experiences of the participants is presented in three sequential phases – a naïve understanding, a structural analysis and a comprehensive understanding. The latter is incorporated within the discussion to avoid repetition.

### Naïve understanding

A preliminary overall interpretation of the narratives emphasised how the four individuals with disabilities were motivated by the human desire to belong. They commenced their interaction with their environments, having a sense of being participants in society, with an expectation that their needs were catered for because of the legislations that gave hope for an inclusive society. Unfortunately, their sense of participation was thwarted by inaccessible environments, which also generated a range of negative emotions and in some cases, a severe decline in well-being. When the environment was accessible, the participants had a heightened sense of belonging and participation. This gave them hope and encouraged them to help make the built environment accessible throughout South Africa.

### Structural analysis

The first step in the second phase involves developing meaning units in the experiences described by the participants. An example is presented in Table 2.

The structural analysis phase is divided into two. The first structural thematic analysis covers the meaning of being in this state of despondency presented (Table 3).

### Becoming discouraged from not being able to participate

For the participants, interacting with the built environment meant developing their sense of belonging in society, being part of a space where they could participate, take an active role and contribute. Each one, however, soon encountered challenges to their full participation. In these moments, they experienced frustration with having to deal with inaccessible environments and other people’s assumptions about disability. They also felt that their disability rights were being violated and became deeply discouraged by inadequate and inappropriate facilities:

‘I began to get a sinking feeling with the way that the staff were treating me. Each of them trying to push me, even though I each time told them that I prefer to roll myself … and have no handles on my wheelchair for that reason’. (Participant D, male, 47 years old)

‘As we got closer to the cellblock building, I saw people walking up the stairs – there was no ramp and I just thought “*oh, no – not now, not here, of all places”*’. (Participant A, male, 42 years old)

### Losing autonomy in the built environment

All the participants experienced subtle but critical moments where their sense of independence within the built environment was lost, and their ability to participate further was threatened. Participants were forced into a situation where they had to carry on struggling on their own or call on others for assistance. This implied that they would have to explain exactly what they needed and how it had to be done. The participants found it cumbersome and exhausting when dealing with people who were not trained or used to dealing with disability. In some instances, getting the assistance they needed was a challenge in itself, and this exacerbated their sense of disempowerment:

‘I get such a headache – all the background noise makes it harder to hear what people are saying and I have to concentrate all the time. So, I approached the security guard and the moment he started speaking I was having trouble lip-reading … the lighting was bad, the shadows from his cap going right across his mouth, and his accent was thick, so I couldn’t work out what he was saying, and I just wanted to get home and lie down’. (Participant C, female, 38 years old)

‘That’s the problem with touch-sensitive (buttons) where you run your finger down lightly over it, a light goes on and the button activates … also, if you’re tactile inclined like me, before you know it you have run your finger over 20 buttons, and then you’re in trouble … because the lift is going up and down like a horse draws and then you’re stuffed. I was in the lift for about 20 minutes waiting for somebody to rescue me, it was late at night and I had just come out of the restaurant’. (Participant B, male, 50 years old)

### Being overcome with anger and humiliation

In some cases, the participant’s sense of injustice, exclusion and loss of dignity was overwhelming and culminated in an overriding state of anger. These were extremely distressing moments where participants felt they had been thrust into a humiliating position by the inaccessible environment, and all focus and unwanted attention was put on their disability. Some also felt ashamed for being the cause of distress to friends and family.

‘So, I reverse down the corridor, and shuffle [along my bottom] off the train, back into my wheelchair … The crowd has reformed, and I squirm in their collective sympathetic looks and comments. They all mean well, but I just need to get out of here. At this stage there are tears rolling down my wife’s face … I am feeling [terrible] for again being the reason for spoiling another nice weekend’. (Participant D, male, 47 years old)

The participants also experienced a feeling of resentment that those responsible lacked accountabilities regarding their duties towards implementing disability policies.

‘The fact that they don’t read the building regulations is not an excuse – they need to read the damn regulations because it is inexcusable that they just don’t bother’. (Participant B, male, 50 years old)

### Feeling despondent and defeated

Reflecting on the status quo and their constant, daily challenges, the participants developed a deep sense of despair. They had all been disabled for many decades and despite witnessing some improvements regarding accessibility, they felt that overall very little had changed since 1994, when South Africa became a democracy.

‘The sad thing is I still cannot move around the city on a normal bus or train – which are pretty obvious forms of transport – so it is difficult not to get despondent about it’. (Participant A, male, 42 years old)

‘It makes me feel very disempowered, I mean this is a system that is well over 100 years old and why on earth should a 53-year-old man not to be able to drive a lift for heaven’s sake.’ (Participant B, male, 50 years old)

In these moments, the participants experienced a sense of being defeated by the environment. They also felt rejected by society and ignored by the government, which intensified their sense of helplessness.

‘You know, you go out and you just want to enjoy yourself and you feel part of something and forget about other stuff like being different – and then a simple thing happens, some little piece of accommodation is missing – and it’s slap bang in your face again and you just feel like giving up’. (Participant A, male, 42 years old)

‘Society in general does not really understand what people with disabilities go through – not really, because even when you explain something, they default back to access ignorance – and it is wilful ignorance because they do not engage.’ (Participant B, male, 50 years old)

Not only the initial structural analysis indicated that the participants experienced discouragement, anger and despondency, but it also revealed their expressions of a desire to be independent. For that reason, a second analysis was undertaken. The essence of the second phase of structural analysis is comprised of the main theme of *Being able to play an active role and contribute to society*, with the following three themes: *Having the desire to participate in society*; *Striving for independence in the built environment;* and *Collaborating to improve accessibility* (Table 4).

### Having the desire to participate

The participants expressed an innate desire to be active in society. During these empowering moments they viewed themselves as regular social beings who interacted with the built environment on an everyday basis, whether they were going to work, enjoying a day out or taking a holiday.

‘And here I was on Robben Island on an accessible bus with a nice big view of everything through the window – it was quite moving, especially spending time at the quarry where Mandela and his fellow prisoners had been forced to work’. (Participant A, male, 42 years old)

‘So, we booked … and paid. We were really excited about the trip; the train is famous all around the world and having the whole train full of our friends promised to be a once in a lifetime experience’. (Participant D, male, 47 years old)

The participants had to maintain a constant awareness of their needs and had become mindful of checking out the environment where possible. Experience had taught them that places were not always accessible as advertised and often they would phone a venue directly to see if it really was accessible, or they would inspect it upon arrival. These instances helped them maintain as much control as they could over their environment.

‘As soon as I check into a hotel I check out the lift, and if I can drive it myself I will – but if I can’t then, even though it irks me, I will just get a bellhop to help me’. (Participant B, male, 50 years old)

Two of the participants held such a strong desire to participate – and to not feel defeated– that they were prepared to put their health at risk. Having come so far in their journey, they wanted to exhaust all the possibilities before throwing in the towel.

‘So … against my wife’s advice, I shuffle (along my bottom) onto the train and lift myself into their narrow wheelchair. OK, at least I am on now…’. (Participant D, male, 47 years old)

‘Well I was determined. So (my care assistant) and I went around the back to see if there was any other way to get in. There were some steel girders stacked against the wall – they were just long enough to use as ramps to go down to the lower level. So, we called some of the other guys to come and help us – a bit scary because the girders were loose, but I was down and along the passages to Nelson Mandela’s cell 46664 – another moving experience’. (Participant A, male, 42 years old)

### Striving for independence in the built environment

The participants stressed that it was not just their ability to be independent that was important. It was also the message that it sent out to other members of society – that people with disabilities could participate, move freely within the built environment and only ask for assistance if it was absolutely necessary.

‘Relying on other people – well you get laconic about it – but I don’t enjoy it. You know I worked really, really hard to be independent. I got a white cane and I’ve got a great dog, and I have the means to get around independently, [*but*] I cannot. [*The bellhops*] are pretty good but that’s not the point, that misses the point of independence, doesn’t it?’ (Participant B, male, 50 years old)

‘So, I carried on for a bit longer and just followed my gut and after a while I saw a sign that went to the right place and was familiar to me – but for a while it was really unpleasant’. (Participant C, female, 38 years old)

### Collaborating to improve accessibility

All four participants expressed a sense of commitment to help improve accessibility in the South African environment. They had spent a lot of their time and energy, both personally and professionally, towards achieving this, and during these moments they believed they were contributing to the increased participation of people with disabilities in general. They felt they had worked hard, over many years, to achieve a level where they could participate in different aspects of society, such as embarking on higher education and avenues of employment. From their involvement in the disability sector, they also believed that enough structures had been created to ensure accommodation of people with disabilities.

‘Considering what the government had pledged – what we have been involved with – I feel I have the right to expect that I can get around on my own because the building regulations require it’. (Participant B, male, 50 years old)

The participants recognised the importance of creating awareness around disability issues at all levels of society and collaborating with the right people in government and private sectors. They carried a deep sense of responsibility towards this and viewed it as an ongoing process.

‘It calls for more awareness – we have to keep on doing what we’re doing and making people aware and calling government to account’. (Participant A, male, 42 years old)

‘I shall write (the luxury train company) a long letter, explaining where they went wrong, and how they need my company’s services to ensure that they comply with their responsibilities as a South African company’. (Participant D, male, 47 years old)

## Discussion

### Comprehensive understanding and reflections

With an extensive knowledge of international and national disability policies and legislations, coupled with the commitment of government to address disability issues (DOP 2013), the participants possibly had a fair and reasonable expectation of inclusion in the South African society to have invested time and resources in the train trip (Participant D), in using available and safe transport facility (Participant C), in visiting a world-renowned tourist site (Participant A) and in using a facility in the built environment to move from one floor to another (Participant B).

However, the findings showed that the participants were caught within a tension of wanting to play an active role within society, but finding it difficult when they tried to participate. Their narrated experiences concurred with previous research that the inclusion of people with disabilities remains a challenge in South African society (Amosun & Taukobong 2010; Amosun et al. 2005; Mayat & Amosun 2011), and particularly within the built environment (Adewumi & Allopi 2014; Lucas 2012; Maart et al. 2007; Napier et al. 2006). These challenges had a profoundly negative impact on their ability to play an active role in society and were detrimental to their quality of life (Hammel et al. 2015; UN 2006).

The WPRPD (DSD 2016) emphasises that accessible infrastructure lies at the core of the right to human dignity, equality and respect for personal space, and the data reiterated that for the disabled, the role of the built environment is critical and goes far beyond the physical realm (DSD 2016; Hammel et al. 2015; Harpur 2012; UN 2006). In other words, denying people with disabilities access to infrastructure severely impedes them from exercising their right to personal mobility, healthcare, employment, education, taking part in cultural life, recreation and sport, political participation, etc. (DSD 2016; Harpur 2012; UN 2006; WHO 2011). To this end, *Part S: Facilities for persons with disabilities* was specifically formulated and incorporated into the *National Building Regulations and Building Standards Act, 1977* (Act No. 103 of 1977) as far back as 1987. This provided specific guidelines to the developers of new infrastructure in South Africa on how to accommodate people with disabilities within the built environment. Furthermore, these regulations were revised in 2011 to keep abreast of, inter alia, a significant increase in the South African population, increasingly complex building control systems, and the introduction of new and innovative construction systems (Watermeyer 2014). Despite these regulations, the experiences of the participants reveal that the current environment significantly inhibits the appreciation of people with disabilities for their diversity and value within society (Clarke et al. 2011).

It emerged that instances of accessibility left the participants feeling energised coupled with a heightened sense of belonging, and this finding supports previous research (Hammel et al. 2015). Therefore, despite the prevailing challenges, it was encouraging that these instances motivated the participants to try overcoming their disappointments and collaborate with other stakeholders in addressing issues of social exclusion for people with disabilities (World Bank 2016).

However, the ongoing lack of policy implementation and sustained inaccessible scenarios ultimately left the participants feeling demoralised and detached from society. The broad range of strong negative emotions generated were similar to the emotions evoked by the lived experiences of adult South African people who stuttered, which ranged from embarrassment to frustration to anger (Klompas & Ross 2004). The manifestation of these emotions may lead to negative behavioural problems and health-related consequences (Tobias & Mukhopadhyay 2017; Krahn et al. 2015; Delbosc & Currie 2011; Klompas & Ross 2004), which may further marginalise people with disabilities from the mainstream of the South African society.

Although most of the findings of the study were generic with respect to those from previous literature, it was felt that the data uncovered uniqueness, particularly as they relate to the South African context, regarding the intensity of the negative impact that environments could exert on people with disabilities. Despite being disabled for over two decades, as well as being from privileged backgrounds, participants were affected to the core of their beings, they were left feeling rejected, inferior, inadequate and questioning their identity within society.

This accentuates once again the magnitude of the role of the built environment in creating an inclusive society.

Furthermore, it is deeply concerning that if these were the experiences of independent, middle-class people with disabilities, there may be little hope for the accommodation of the majority of the disabled population, who are indigent and lack resources (Statistics South Africa 2011). The *Constitution* of South Africa enshrines the right of everyone to an ‘environment that is not harmful to their health or well-being’ (Section 24 Bill of Rights 1996). Hence, the data indicate a failure of government and other stakeholders to address the protection, safety and general needs of the most neglected groups within the scope of disability (DSD 2016; UN 2006, 2015). Furthermore, the lived experiences of the participants revealed that implementation challenges to disability inclusion prevail at an attitudinal, environmental and institutional level (Harpur 2012). The slow delivery of policy and legislation also implies that the target goals identified in the Sustainable Development Goals (SDGs) (UN 2015), NDP (DOP 2013) and the WPRPD (DSD 2016) will not be reached in the allotted timeframes.

In turn, this suggests that new infrastructure will continue to be developed in an inaccessible manner, thereby perpetuating the marginalisation of people with disabilities into the next generation (McKinney 2016). Overall, the findings call for urgent strategies to address current and future implementation of policy, as well as increased involvement of relevant stakeholders (Hammel et al. 2015) including people with disabilities themselves (DSD 2016; McKinney 2016; UN 2006). Finally, more research is needed across a broader range of disabilities to examine the lived experience of people with disabilities within the broader South African built environment.

### Limitations of the study

The profiles of the four participants in this study do not reflect the diversity in the population of South Africa (Maart et al. 2007). All the participants were white, middle-class, with postgraduate academic qualifications. Therefore, the findings of the study are not generalisable as the majority of people with disabilities in South Africa are black, poorly educated and from a lower socio-economic background, being reliant on disability grants for survival (Maart et al. 2007). Similarly, it is acknowledged that the examples of the built environment used in the study are more recognised as high-end forms of commuter travel in South Africa that are not affordable to most of the population.

## Conclusion

In conclusion, this study agrees that an accessible built environment is an essential step towards creating an inclusive society. The findings of this study provide a deeper understanding of the experiences of people with disabilities, who are driven by a desire to take part and be independent, and through this process develop their sense of belonging and dignity within society. Despite existing policies to ensure that people with disabilities are accommodated, barriers to participation prevail, which are detrimental to quality of life and well-being and have a negative impact on the possibility of future participation.

## Appendix 1: Interview schedule with the person with a disability.

1. How has South Africa embraced a disability-friendly environment?
2. How successful has the policy been?
3. Do you think reasonable accommodation is a fair requirement in any community?
4. Is it a burden?
5. What would the person with a disability expect?
6. Is employing a person with a disability a risk (financial, more work)? Is it worth entertaining this risk and how can this risk be minimised?
7. Would there be positive or motivating factors to accommodate people with disabilities in this community? (If you imagine a community in which people with disabilities are fully integrated and accommodated, what would make it a positive community to live in?)
8. How would you define disability?
9. In your experience what has been the impact of interaction of people with disabilities?
10. Could you please describe an experience that you have had within the South African built environment? Could you please describe the incident in greater detail? How did it make you feel?
11. What, if anything, needs to happen or change to allow people with disabilities to be accommodated within the community?
12. Do you have any opinion or experience with [the university] – How has it embraced a disability-friendly environment?
13. In the study, the Disability Sector is regarded as the ‘consumer’ – in a sense that it is on the receiving end of what [the university] produces as students. The study is exploring how [the university] is preparing its students to contribute to an inclusive society.
  13.1 What approach would you expect the university to adopt to achieve that?
  13.1 What resources do you think they have?
  13.1 What barriers/challenges do they face?
14. With regard to engineering as a discipline, do you think they should have knowledge on disability incorporated into their curriculum? In what way? What would you expect?
